# Excellent resistive switching properties of atomic layer-deposited Al_2_O_3_/HfO_2_/Al_2_O_3_ trilayer structures for non-volatile memory applications

**DOI:** 10.1186/s11671-015-0846-y

**Published:** 2015-03-19

**Authors:** Lai-Guo Wang, Xu Qian, Yan-Qiang Cao, Zheng-Yi Cao, Guo-Yong Fang, Ai-Dong Li, Di Wu

**Affiliations:** National Laboratory of Solid State Microstructures and Department of Materials Science and Engineering, College of Engineering and Applied Sciences, Collaborative Innovation Center of Advanced Microstructures, Nanjing University, 22 Hankou Road, Nanjing, 210093 People’s Republic of China; Anhui Key Laboratory of Functional Coordination Compounds, School of Chemistry and Chemical Engineering, Anqing Normal University, 128 Linghu South Road, Anhui, 246011 People’s Republic of China

**Keywords:** Non-volatile memory, Atomic layer deposition, Resistance random access memory, Trilayer structure

## Abstract

We have demonstrated a flexible resistive random access memory unit with trilayer structure by atomic layer deposition (ALD). The device unit is composed of Al_2_O_3_/HfO_2_/Al_2_O_3_-based functional stacks on TiN-coated Si substrate. The cross-sectional HRTEM image and XPS depth profile of Al_2_O_3_/HfO_2_/Al_2_O_3_ on TiN-coated Si confirm the existence of interfacial layers between trilayer structures of Al_2_O_3_/HfO_2_/Al_2_O_3_ after 600°C post-annealing. The memory units of Pt/Al_2_O_3_/HfO_2_/Al_2_O_3_/TiN/Si exhibit a typical bipolar, reliable, and reproducible resistive switching behavior, such as stable resistance ratio (>10) of OFF/ON states, sharp distribution of set and reset voltages, better switching endurance up to 10^3^ cycles, and longer data retention at 85°C over 10 years. The possible switching mechanism of trilayer structure of Al_2_O_3_/HfO_2_/Al_2_O_3_ has been proposed. The trilayer structure device units of Al_2_O_3_/HfO_2_/Al_2_O_3_ on TiN-coated Si prepared by ALD may be a potential candidate for oxide-based resistive random access memory.

## Background

With traditional memories approaching their scaling limit, new memory concepts and materials in ultra-large-scale integration have drawn much attention. Resistive random access memory (RRAM) is one of the most promising candidates for next-generation non-volatile memory applications due to its simple structure, low power consumption, high-speed operation, nondestructive readout, and high-density integration [1]. Many semiconducting and insulating materials including binary transition metal oxides, perovskite oxides, chalcogenides, sulfides, amorphous silicon, organic materials, and ferroelectric materials have been investigated extensively for RRAM applications [2], especially metal oxides such as Pr_1 − *x*_Ca_*x*_MnO_3_ [3,4], SrZrO_3_ [5], STO [6], Nb_2_O_5_ [7], NiO [8], ZrO_2_ [9], SiO_2_ [10], WO_3_ [11], TiO_2_ [12,13], Al_2_O_3_ [14], ZnO [15], and HfO_2_ [16-18].

However, devices using metal oxides suffer from the dispersion of resistive switching parameters, such as the resistance values of low and high resistance states (LRS and HRS), the required set voltages from the HRS to the LRS, and reset voltages from the LRS to the HRS, which may lead to false programming and readout error [19]. Ruptures of the conducting filaments with various sizes at random locations are thought as the main reason for the non-uniformity of resistive switching parameters [20,21]. Several methods have been attempted to solve this problem, such as minimizing grain boundaries, doping, embedding nanoparticles, device area scaling, and bilayer structures in the memory devices [22-28]. The bilayer structure devices have been confirmed with evidently improved resistive switching behaviors [24,25]. Several models have been proposed to explain the enhanced performance [29,30]. Moreover, shrinkage of the active memory unit area to sub-100-nm size using a plug-contact-type bottom electrode was also able to obtain a sharp distribution in switching parameters.

The atomic layer deposition (ALD) is a kind of unique and modified chemical vapor deposition (CVD) method [31,32]. The precursor vapors are pulsed into the reactor alternatively and separated by purging with an inert gas. This results in a stepwise surface-saturated and self-limiting gas-solid reaction mechanism with many advantages such as good reproducibility, excellent conformity and uniformity over large area, low deposition temperature, and simple and precise control of film thickness, especially for deposition of nano-laminated structure. In this work, we fabricated trilayer-structure flexible RRAM units based on Al_2_O_3_/HfO_2_/Al_2_O_3_ functional stacks on TiN-coated Si substrate by ALD so as to achieve excellent resistive switching performances with negligible parameter dispersion.

## Methods

**Figure 1:**
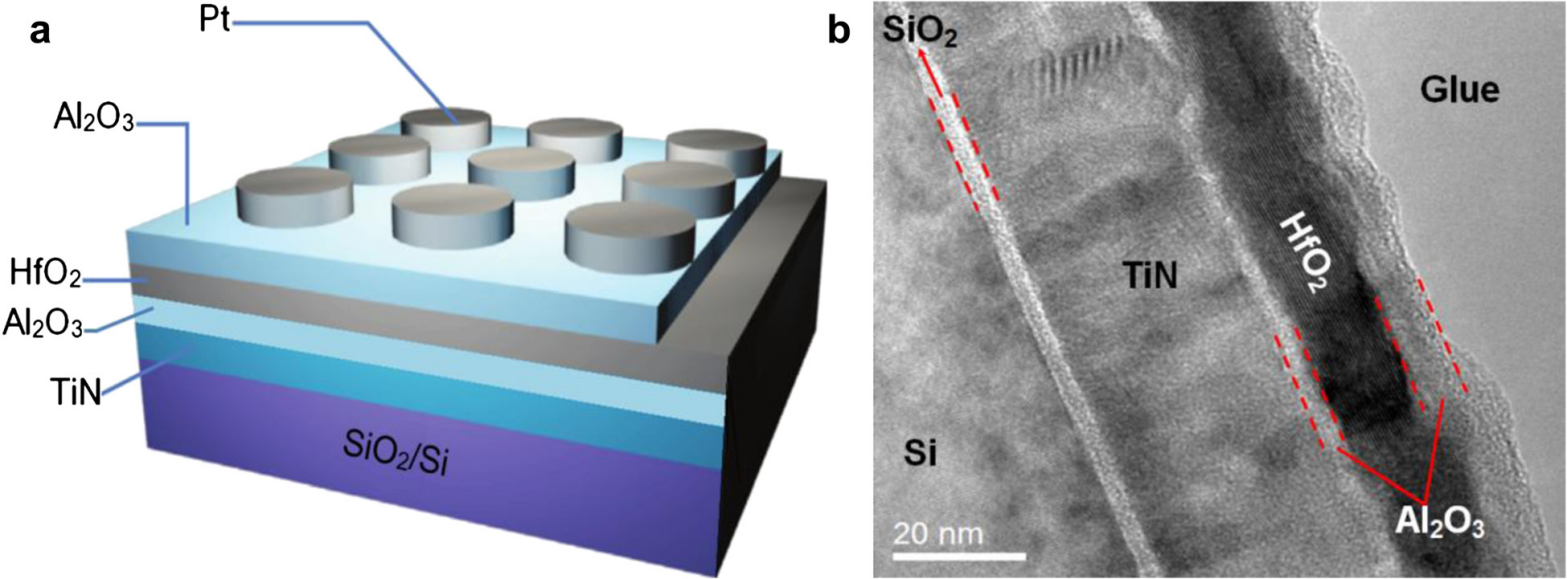
**RRAM device structures and device unit of Al**_**2**_ **O**_**3**_ **/HfO**_**2**_ **/Al**_**2**_ **O**_**3**_ **trilayer structure. (a)** Schematic and test configuration of the RRAM device structures of Al_2_O_3_/HfO_2_/Al_2_O_3_ trilayer structure on TiN-coated Si with Pt top electrode. **(b)** Typical cross-sectional TEM image of the device unit of Al_2_O_3_/HfO_2_/Al_2_O_3_ trilayer structure on TiN-coated Si by ALD.

ALD was performed in a commercial Picosun SUNALE^TM^ R-200 advanced reactor (Picosun, Masalantie 365, FI-02430 Masala, Finland). P-type Si (100) wafers with a resistivity of 1 ~ 10 Ω · cm were used as the starting substrates. After the conventional RTA cleaning of the Si wafers without removing native oxide with the diluted HF solution, 30-nm-thick TiN was deposited on Si as bottom electrode at 400°C using TiCl_4_ at room temperature (RT) and NH_3_ plasma gas as the Ti and N sources by plasma-enhanced atomic layer deposition (PEALD). Liquid NH_3_ at room temperature was used as NH_3_ plasma source. The plasma power and NH_3_ gas flow rate were 2,500 W and 150 sccm, respectively. Subsequently, 6 nm Al_2_O_3_/10 nm HfO_2_/3 nm Al_2_O_3_ stacking structures (Figure 1a) were deposited in turn on TiN-coated Si substrates at 250°C by thermal ALD using Hf[N(C_2_H_5_)CH_3_]_4_ (TEMAH), Al(CH_3_)_3_, and H_2_O as the Hf, Al, and O sources, respectively, where one oxide cycle consisted of 0.1-s metal source injection, 4-s N_2_ purging, 0.1-s H_2_O injection, and 4-s N_2_ purging. TEMAH was evaporated at 150°C. Pure N_2_ (99.999%) was used as carrier gas and purge gas. Then, 100-nm-thick Pt top electrodes were DC sputtered through a shadow mask with a diameter of 150 μm using the Q150T system. Post-metallization annealing (PMA) was performed at 600°C for 60 s in N_2_ using rapid thermal annealing so as to remove slight organic residue in oxide stacking and improve the ohmic contact between the electrodes and metal oxide films.

The growth per cycle (GPC) of pure Al_2_O_3_ or HfO_2_ on Si was determined by spectroscopic ellipsometer (GES-5, Annecy-le-Vieux, France). The nominal thickness of Al_2_O_3_/HfO_2_/Al_2_O_3_ trilayer structure by ALD on TiN-coated Si was evaluated to be about 6.1, 10.3, and 3.0 nm, respectively. The actual thickness and microstructures of Al_2_O_3_/HfO_2_/Al_2_O_3_ on TiN-coated Si were examined by a transmission electron microscope (TEM, Tecnai G2F20 S-Twin, FEI, Hillsboro, OR, USA) operating at 200 kV. The composition and chemical state of the samples were analyzed via X-ray photoelectron spectroscopy (XPS, Thermo Fisher K-Alpha, Thermo Fisher Scientific, Waltham, MA, USA) with a monochromatic Al K_α_ source (*hν* = 1,486.6 eV) for excitation of photoelectrons. The charge effects were calibrated by setting the C 1-s photoemission at 284.6 eV. The XPS depth profile of Al_2_O_3_/HfO_2_/Al_2_O_3_ on TiN-coated Si was obtained by Ar ion etching. The resistive switching properties were measured under different modes using a Keithley 4200-SCS semiconductor characterization system at room temperature and 85°C. A current compliance of 10 mA was imposed to protect the fabricated device units from damages of high currents during set processes.

## Results and discussion

The schematic of the RRAM device structures of Al_2_O_3_/HfO_2_/Al_2_O_3_ trilayer structure by ALD on TiN-coated Si with Pt top electrode is illustrated in Figure 1a. Figure 1b shows the typical cross-sectional TEM image of the device unit. The laminated structures of Al_2_O_3_/HfO_2_/Al_2_O_3_/TiN/Si have been recognized clearly with the native oxide layer of SiO_2_ between TiN and Si substrate. The measured average thickness of Al_2_O_3_/HfO_2_/Al_2_O_3_/TiN/SiO_2_ on Si is 6.1, 13.0, 3.0, 30.2, and 3.3 nm, respectively, which is basically consistent with the prescribed ones. After 600°C PMA, the partial and complete crystallization in TiN bottom electrode and HfO_2_ interlayer with two Al_2_O_3_ amorphous layers is observed, respectively. Meanwhile, the Al_2_O_3_/HfO_2_/Al_2_O_3_ trilayer structure on TiN-coated Si shows relatively rough interface and surface due to the crystallization of HfO_2_ and TiN interlayers or possible interfacial diffusion between various layers at 600°C.

**Figure 2:**
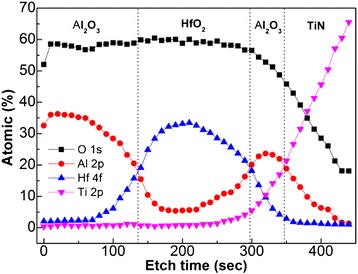
**XPS depth profile of Al**_**2**_ **O**_**3**_ **/HfO**_**2**_ **/Al**_**2**_ **O**_**3**_ **on TiN-coated Si by Ar ion etching.**

Figure 2 shows the XPS depth profile of Al_2_O_3_/HfO_2_/Al_2_O_3_ on TiN-coated Si by Ar ion etching. The trilayer structure of Al_2_O_3_/HfO_2_/Al_2_O_3_ on TiN-coated Si can be seen. Moreover, the significant interfacial diffusion between Al_2_O_3_/HfO_2_ and Al_2_O_3_/TiN has been also confirmed, in good agreement with the TEM cross-sectional image. In spite of this, the memory units of Al_2_O_3_/HfO_2_/Al_2_O_3_ trilayer structure on TiN-coated Si have been fabricated by ALD successfully.

**Figure 3:**
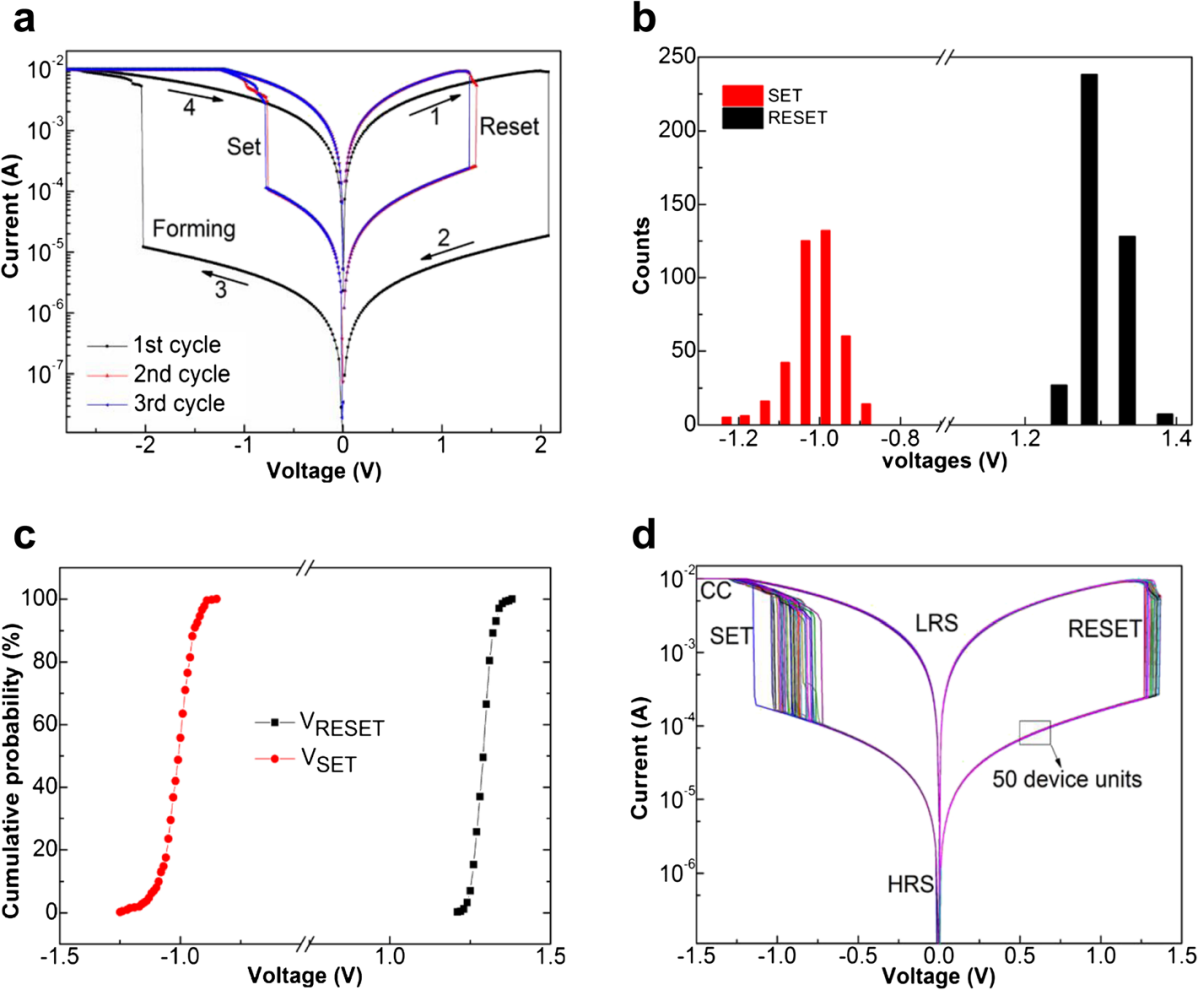
**Resistive switching characteristics of the device unit and distribution of the set and reset voltages. (a)** Typical resistive switching characteristics of the device unit of Pt/Al_2_O_3_/HfO_2_/Al_2_O_3_/TiN/Si after initial, second, and third cycles. **(b, c)** Statistical results of distribution and cumulative probability of the set and reset voltages measured from a device unit for 400 times tests. **(d)** Resistive switching data of 50 randomly selected device units.

The I-V curves of the device unit of Pt/Al_2_O_3_/HfO_2_/Al_2_O_3_/TiN/Si with various cycles are plotted in Figure 3a, indicating a typical bipolar resistive switching characteristic. For almost all the samples, larger forming voltage is needed to form conductive channels before the switching test. The forming voltage of device unit is about −2 V. The initial resistance state of the memory unit in the first cycle (black curve) is higher than that of the second and third cycles (blue and red curves). Moreover, as-prepared original device unit is in the LRS and an excess positive voltage of 2 V is needed to reset the device unit from LRS to HRS (denoted by arrows 1 and 2 in Figure 3a). The I-V curves from the second and third cycles are almost in superposition with similar set and reset voltages.

For high-density memory application, uniformity of both set and reset voltages is very important. Figure 3b,c plots the statistical results of distribution and cumulative probability of the set and reset voltages measured from a single device unit for 400 times tests. The set and reset voltages show narrow distribution from −1.21 to −0.85 V and from 1.24 to 1.38 V in a device unit, respectively. Their corresponding average values are −0.96 and 1.31 V, respectively. Moreover, 50 randomly selected device units also exhibit less deviation of set and reset voltages, as seen in Figure 3d. Especially, the reset voltage has better monodispersion than the set voltage in Figure 3b-d.

**Figure 4:**
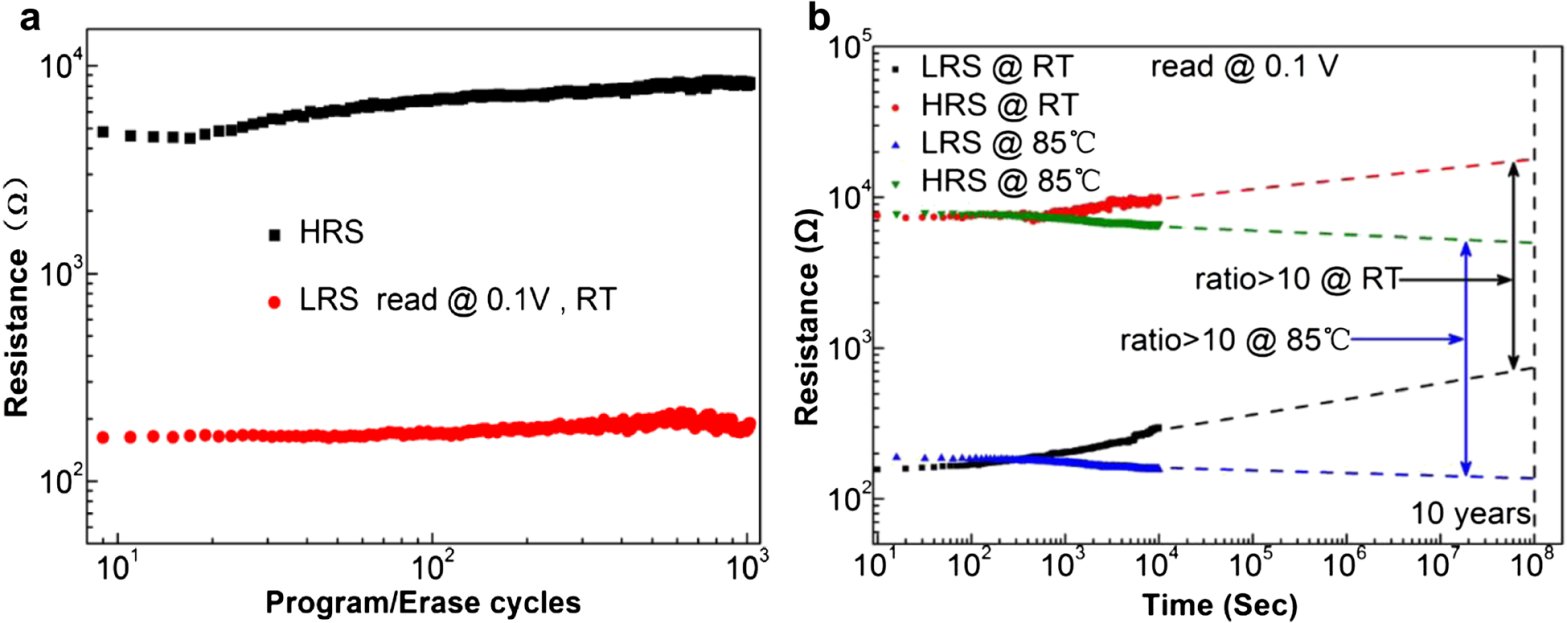
**The durability of the device unit of Pt/Al**_**2**_ **O**_**3**_ **/HfO**_**2**_ **/Al**_**2**_ **O**_**3**_ **/TiN/Si. (a)** The continuous program and erase test. **(b)** Read disturbance test for device after 10^4^-s retention time at room temperature and 85°C.

The switching endurance and data retention characteristics of the device unit of Pt/Al_2_O_3_/HfO_2_/Al_2_O_3_/TiN/Si have been examined. The stable and reproducible switching properties have been achieved in Figure 4a,b. The sweeping voltage was applied from 0 to −2 V for set and 0 to 1.5 V for reset with a reading voltage of 0.1 V at room temperature. The endurance test shows a consistent 10^3^ switching cycle with a stable resistance ratio of OFF/ON states above 10 (Figure 4a). Retention characteristics of the device unit were measured at room temperature and 85°C, as seen in Figure 4b. Both HRS and LRS were read at 0.1 V for cumulative waiting time of 10^4^ s. At room temperature, no obvious degradation of the memory window is observed with slightly increasing LRS and HRS. At 85°C, the memory window exhibits better thermal stability with the resistance ratio of LRS/HRS >10, indicating a better retention endurance of the memory unit over a 10-year lifetime based on the extrapolation method. It is interesting that in the retention test, both HRS and LRS increase with time at room temperature but decrease at 85°C on the contrary. This phenomenon can be explained by the change of oxygen vacancy concentration with time at room temperature and 85°C. As known, the oxygen vacancy concentration of some oxide thin film samples stored in air ambient is not constant [33]. The air oxygen may slowly diffuse into the Al_2_O_3_/HfO_2_/Al_2_O_3_-based samples at room temperature, leading to the gradual decrease of oxygen vacancy concentration, i.e. the increase of HRS and LRS in oxide thin films with retention time. When raising the measuring temperature to 85°C, on one hand, the oxygen diffusion into the device unit reduces the oxygen vacancy concentration; on the other hand, the enhanced temperature may produce more oxygen vacancies in Al_2_O_3_/HfO_2_/Al_2_O_3_ trilayer structures. Evidently the increased carriers of oxygen vacancies predominate, so both HRS and LRS of the device unit decrease with time at 85°C.

Evidently, the above results confirm that the device unit of trilayer structure of Al_2_O_3_/HfO_2_/Al_2_O_3_ on TiN-coated Si exhibits excellent resistive switching performances, such as stability of resistance ratio of on/off states, uniformity of set and reset voltages, and reliability and durability of switching between the LRS and the HRS.

In order to obtain in-depth understanding on switching mechanism of trilayer structure of Al_2_O_3_/HfO_2_/Al_2_O_3_ on TiN-coated Si, we performed the XPS analyses on Al_2_O_3_/HfO_2_/Al_2_O_3_ structures. XPS spectra were fitted with Gaussian-Lorentzian (G-L) functions after smart-type background subtraction.

**Figure 5:**
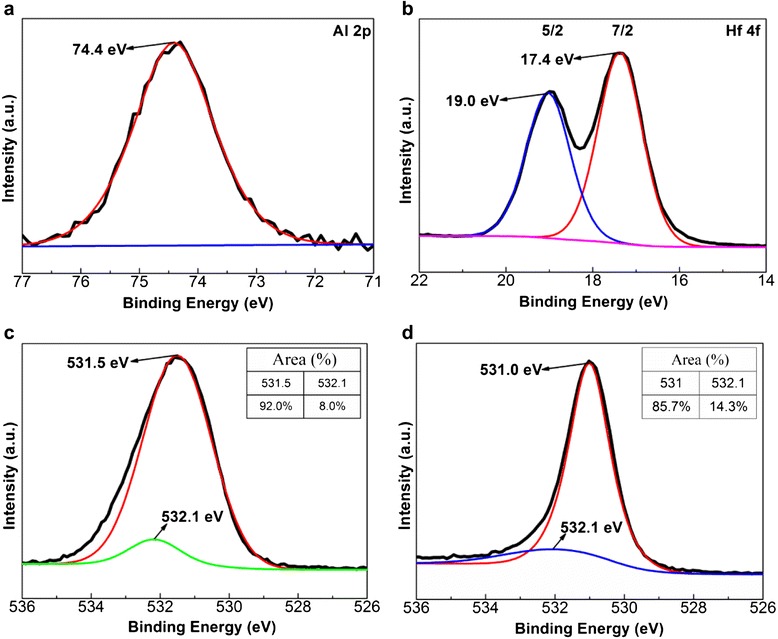
**Narrow-scan XPS spectra from trilayer structure of Al**_**2**_ **O**_**3**_ **/HfO**_**2**_ **/Al**_**2**_ **O**_**3**_ **on TiN-coated Si. (a)** Al 2p, **(b)** Hf 4f peaks of Al_2_O_3_/HfO_2_/Al_2_O_3_ O 1-s peaks of **(c)** Al_2_O_3_, and **(d)** HfO_2_ layers.

The narrow-scan XPS spectra of Al 2p, Hf 4f, and O 1-s peaks in Al_2_O_3_ and HfO_2_ layers are shown in Figure 5a-d. The Al 2p peak is located at 74.4 eV, which is assigned to Al-O bonding. The Hf 4f_5/2_ and Hf 4f_7/2_ peaks at 19.0 and 17.4 eV with a spin-orbit splitting of 1.6 eV are consistent with the literature data of high *k* HfO_2_/Si [34]. The O 1-s spectra from Al_2_O_3_ and HfO_2_ layers can be deconvoluted into two peaks in Figure 5c,d. The slightly lower binding energies of the O 1-s peak at around 531.5 and 531.0 eV, which correspond to Al-O and Hf-O bonding in Al_2_O_3_ and HfO_2_ layers, respectively. Whereas the slightly higher energy of 532.1 eV in the O 1-s spectra of Figure 5c, d is attributed to the oxygen vacancies in Al_2_O_3_ and HfO_2_ layers based on the literature reports [35,36]. The inset tables in Figure 5c, d list the area proportion of each peak. The percentage of oxygen vacancies in the Al_2_O_3_ and HfO_2_ layer is about 8.0% and 14.3%, respectively. Evidently, the oxygen vacancy concentration of HfO_2_ is higher than that of Al_2_O_3_. The TEM and XPS depth results of trilayer structure of Al_2_O_3_/HfO_2_/Al_2_O_3_ confirm the existence of significant interfacial diffusion between Al_2_O_3_ and HfO_2_ films. Defect equation of the interfacial diffusion can be expressed as:

1 $$ \mathrm{A}{\mathrm{l}}_2{\mathrm{O}}_3\overset{{\mathrm{Hf}\mathrm{O}}_2}{\to }2\mathrm{A}{\mathrm{l}}_{\mathrm{Hf}}^{\prime }+3{\mathrm{O}}_{\mathrm{O}}^{\times }+{{\mathrm{V}}_{\mathrm{O}}}^{\cdot \cdot } $$

2 $$ {\mathrm{Hf}\mathrm{O}}_2\overset{{\mathrm{Al}}_2{\mathrm{O}}_3}{\to }2{\mathrm{Hf}}_{\mathrm{Al}}^{.}+2{\mathrm{O}}_{\mathrm{O}}^{\times }+{\mathrm{O}}_i^{{\prime\prime} } $$

Where ${\mathrm{Al}}_{\mathrm{Hf}}^{\prime }$ means that Al^3+^ occupies the position of Hf^4+^ in HfO_2_ lattice, with singular negative charge. *V*_O_^⋅ ⋅^ refers to oxygen vacancy with double positive charge in oxide lattice. ${\mathrm{Hf}}_{\mathrm{Al}}^{.}$ means that Hf^4+^ occupies the position of Al^3+^ in Al_2_O_3_ lattice, with singular positive charge. ${\mathrm{O}}_i^{{\prime\prime} }$ means oxygen anion on an interstitial site, with double negative charge. ${\mathrm{O}}_{\mathrm{O}}^{\times }$ represents the neutral oxygen atom in an oxide lattice site.

**Figure 6:**
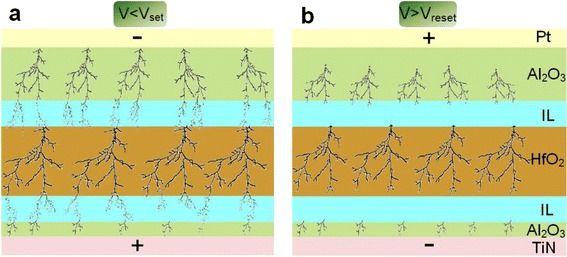
**Schematics of proposed filament-switching mechanism of Pt/Al**_**2**_ **O**_**3**_ **/HfO**_**2**_ **/Al**_**2**_ **O**_**3**_ **on TiN-coated Si. (a)** Low resistance state (LRS) when applied voltage is less than set voltage. **(b)** High resistance state (HRS) when applied voltage is greater than reset voltage.

Therefore, based on Equations 1 and 2, HfO_2_ layer produces and stores more oxygen vacancies than Al_2_O_3_ layer. The physical size of the conductive filament in oxygen vacancy-deficient layer is narrower than in the oxygen vacancy-rich layer [19], so it can be inferred reasonably that the filament in HfO_2_ layer is thicker and more intensive. Based on the bilayer-structure model, the oxygen exchange through the interface between two layers played an important role in improving resistive switching characteristics [19]. In this work, the two interfacial layers (IL) between trilayer-structure of Al_2_O_3_/HfO_2_/Al_2_O_3_ on TiN-coated Si have also a similar effect on connected or broken filaments in memory units, as indicated in Figure 6a,b.

The two interfacial layers between Al_2_O_3_/IL/HfO_2_/IL/Al_2_O_3_ could affect the growth position, direction, and overlapping of conductive filaments. The linkage (Figure 6a) or rupture (Figure 6b) of the conductive filaments corresponds to the SET from HRS to LRS or the RESET from LRS to HRS, respectively, which more easily occur in two interfacial layers. Further, the shape and position of the conductive filaments in Al_2_O_3_ and HfO_2_ layers change less in the SET and RESET processing. The resistive switching mechanism is mainly dominated by formation and rupture of conducting filaments of oxygen vacancies in interfacial layers. The thermal redox near the interface between the oxides or electrochemical migration of oxygen ions may be also the main driving force [4,37,38]. Further work is needed to understand this mechanism well.

## Conclusions

In summary, reliable and uniform RRAM units based on trilayer structure of Al_2_O_3_/HfO_2_/Al_2_O_3_ on TiN-coated Si have been prepared by ALD. The cross-sectional HRTEM image and XPS depth profile of Al_2_O_3_/HfO_2_/Al_2_O_3_ on TiN-coated Si confirm the existence of interfacial layers between the trilayer structure of Al_2_O_3_/HfO_2_/Al_2_O_3_. The memory units of Pt/Al_2_O_3_/HfO_2_/Al_2_O_3_/TiN/Si exhibit a typical bipolar, reliable, and reproducible resistive switching behavior, such as stable resistance ratio (>10) of OFF/ON states, sharp distribution of set and reset voltages, better switching endurance up to 10^3^ cycles, and longer data retention at 85°C over 10 years. The possible switching mechanism of trilayer structure of Al_2_O_3_/HfO_2_/Al_2_O_3_ has been proposed, and two interfacial layers between Al_2_O_3_/IL/HfO_2_/IL/Al_2_O_3_ play an important role in improving resistive switching characteristics. The oxygen ion migration and the redox reactions at the tip of the localized filament lead to the formation/rupture of the conducting filaments mainly in two interfacial layers during the SET and RESET processing. The Al_2_O_3_/HfO_2_/Al_2_O_3_ trilayer structure device units by ALD may be a potential candidate for oxide-based RRAM.
